# Evaluation of causal associations between interleukin-18 levels and immune-mediated inflammatory diseases: a Mendelian randomization study

**DOI:** 10.1186/s12920-023-01744-z

**Published:** 2023-11-29

**Authors:** Jialing Wu, Xi Zhang, Dongze Wu, Ou Jin, Jieruo Gu

**Affiliations:** 1 Department of Rheumatology and Immunology, Third Affiliated Hospital of Sun Yat-Sen University, 600 Tianhe Road, Guangzhou, 510630 China; 2 Department of Rheumatology and Immunology, Sichuan Provincial People’s Hospital, University of Electronic Science and Technology of China, Chengdu, China

**Keywords:** Interleukin-18, Immune-mediated inflammatory diseases, Mendelian randomization, Systemic lupus erythematosus, Inflammatory bowel disease

## Abstract

**Background:**

Altered interleukin (IL)-18 levels are associated with immune-mediated inflammatory diseases (IMIDs), but no studies have investigated their causal relationship. This study aimed to examine the causal associations between IL-18 and IMIDs.

**Methods:**

We performed a two-sample Mendelian randomization (MR) analysis. Genetic variants were selected from genome-wide association study datasets following stringent assessments. We then used these variants as instrumental variables to estimate the causal effects of IL-18 levels on the risk of developing five common IMIDs: rheumatoid arthritis (RA), systemic lupus erythematosus (SLE), inflammatory bowel disease (IBD), ankylosing spondylitis (AS), and psoriasis. We used the inverse variance-weighted (IVW) method as the primary analysis, with sensitivity analyses performed to avoid potential bias. Reverse-direction MR analyses were performed to rule out the possibility of reverse associations.

**Results:**

We found that genetically determined higher circulating IL-18 levels were causally associated with a higher risk for SLE (*P*_*IVW*_ = 0.009; OR, 1.214; 95% CI, 1.049 − 1.404) and IBD (*P*_*IVW*_ < 0.001; OR, 1.142; 95% CI, 1.062 − 1.228), but found no significant associations of IL-18 with RA (*P*_*IVW*_ = 0.496; OR, 1.044; 95% CI, 0.923 − 1.180), AS (*P*_*IVW*_ = 0.021; OR, 1.181; 95% CI, 1.025 − 1.361), or psoriasis (*P*_*IVW*_ = 0.232; OR, 1.198; 95% CI, 0.891 − 1.611). In the reverse direction, no causal relationship existed between SLE or IBD and IL-18 levels. Globally, sensitivity studies using alternative MR methods supported the results that were robust and reliable. The Cochran’s Q test, MR-Egger intercept, and MR-Pleiotropy RESidual Sum and Outlier excluded the influence of heterogeneity, horizontal pleiotropy, and outliers.

**Conclusions:**

We have demonstrated that elevated IL-18 levels increase the risk of SLE and IBD but not RA, AS, or psoriasis. The results enhanced our understanding of IL-18 in the pathology of IMIDs.

**Supplementary Information:**

The online version contains supplementary material available at 10.1186/s12920-023-01744-z.

## Background

Immune-mediated inflammatory diseases (IMIDs) are a collection of common, chronic, and complex disorders with diverse clinical manifestations. These diseases, characterized by dysregulation of the immune system and imbalance of target organ inflammatory cytokines, can lead to severe morbidity, decreased quality of life, and premature death [1]. Examples include but are not limited to rheumatoid arthritis (RA), systemic lupus erythematosus (SLE), inflammatory bowel disease (IBD), ankylosing spondylitis (AS), and psoriasis (PsO), most of which are currently incurable. While treatments exist to control symptoms and slow disease progression, ongoing research is exploring new personalized therapies, such as targeted biologics, to provide more effective treatment options. There are public and private hierarchical immune pathways driving pathogenesis across IMIDs, which may be related to treatment efficacy. On one hand, the successful use of tumour necrosis factor (TNF) inhibitors in a range of IMIDs has clarified the concept of a common pathway. On the other hand, the experience of IL-6 receptor and IL-6 inhibition succeeding in RA and vasculitis while failing in PsO and axial spondylarthritis suggests the existence of private pathways [2]. Withstanding this situation, using cytokine-based classification can better address pathophysiological commonalities and substantial mechanistic differences among IMIDs, thus potentially assisting in development of effective and targeted therapies [3].

Interleukin (IL)-18, a pro-inflammatory cytokine, is an essential mediator in innate and adaptive immune responses and is thought to have unignorable roles in several IMIDs. As a member of the IL-1 superfamily, IL-18 is produced mainly by monocytes/macrophages and dendritic cells [4]. IL-18, in combination with IL-12, induces B cells and T helper 1 (Th1) cells to produce interferon (IFN) -γ, which triggers a Th1 immune response [5]. Dysregulation of IL-18 levels may lead to autoimmune or inflammatory diseases involving host defence, cancer, allergy, immune response, arthritis, etcetera [4]. Previous reports have implicated its role in the pathogenesis of IMIDs. Elevated levels of IL-18 were found in situ of the lesion or in the peripheral circulatory system, including the synovial fluid of patients with RA [6], the skin of patients with PsO [7], the intestinal samples of patients with IBD [8], the serum and glomeruli of patients with SLE [9], and the serum of patients with AS [10]. Blocking IL-18 is effective in attenuating disease damage in animal studies, including dextran sulphate sodium-induced colitis [11], collagen-induced model of arthritis [12], and nephritis of MRL/lpr mice [13]. Nevertheless, whether IL-18 works as a common downstream effector or a signature cytokine hub for these diseases remains unknown. Targeting IL-18 as a future therapeutic approach for IMIDs requires more evidence. Therefore, clarifying the causal role of IL-18 on these IMIDs can be meaningful for elucidating disease pathogenesis and further drug development.

Mendelian randomization (MR) offers an opportunity to provide causal evidence that cannot be obtained adequately from conventional observational studies or is not amenable to evaluation when randomized controlled trials (RCTs) (interventional trials) are unethical or illogical to conduct [14]. Considered a natural analogue of the classical RCTs, MR usually applies genetic variants from publicly available summary-level data as instrumental variables (IVs) to investigate the causality of a relationship between an exposure and an outcome [15]. The growing availability of data and the vast expansion of methods enable MR to be a popular, feasible, and economical approach for evaluating causal associations [14]. In this study, we used two-sample MR to assess causal associations between circulating IL-18 levels and the risk of several IMIDs, including RA, SLE, IBD, AS, and PsO.

## Methods

### Study design

In brief, we conducted a two-sample MR study to evaluate the causal associations of IL-18 levels on five IMIDs (RA, SLE, IBD, AS, and PsO) using associated single-nucleotide polymorphisms (SNPs) as IVs. The MR study is only conducted when genetic instruments meet following three assumptions: first, genetic variants must be robustly associated with exposure; second, genetic variants should not be related to potential confounders of the exposure-outcome relationship; third, genetic variants cannot affect outcome risk through any alternative way but only through exposure. The overall study followed STROBE-MR (Strengthening the reporting of observational studies in epidemiology using mendelian randomization) guideline [16, 17] (Supplementary Table S1).

### Data resources

The MR analysis in this study was based on previously published independent genome-wide association studies (GWASs), from which we obtained summarized level data (effect size estimates and corresponding standard errors) on the associations between genetic variants and exposure (IL-18) or outcome (RA, SLE, IBD, AS, and PsO). In the current study, the detailed data for IL-18 came from a GWAS in 14,744 healthy subjects of European ancestry, with multiple common genetic variants that influence circulating cytokine levels identified [18]. The data for RA was gathered from a GWAS involving 17,221 cases and 74,823 controls of European ancestry [19]. The RA cases fulfilled the 1987 American College of Rheumatology (ACR) criteria or the 2010 ACR/European League Against Rheumatism criteria, or were diagnosed with RA by a professional rheumatologist. The data used for SLE was obtained from a GWAS that included 14,267 individuals of European ancestry, comprising 5,201 cases and 9,066 controls, and the cases were defined based on standard ACR classification criteria [20]. For IBD, we used summary statistics from a GWAS of the UK Biobank involving European individuals (7,045 self-reported cases and 456,327 controls) [21]. The psoriasis data was obtained from a GWAS that included 13,229 psoriasis cases diagnosed by dermatologists and 21,543 controls [22]. For AS, the data were obtained from the R9 release of the FinnGen consortium, including 2,860 AS cases and 270,964 controls. In the FinnGen research project, AS cases were identified and defined based on the International Classification of Disease-10 code (M13) with diagnostic information obtained from national health registries in Finland [23]. Information on all datasets was listed in Supplementary Table S2.

### Selection of genetic instruments

The SNPs that influence circulating concentrations of IL-18 were selected at a significance threshold of a *P* value < 5 × 10^–8^ and would be used as IVs. We performed linkage disequilibrium (LD)-pruning on the selected SNPs, only retaining the SNP with the lowest *P* value as independent IVs (with an *r*^*2*^ value of 0.1 and clumping distance of 10,000 kb). All these IVs were verified for sufficient strength by evaluating the *F*-statistics (*F* > 10), and no pleiotropic instruments were identified as assessed using the Phenoscanner (http://www.phenoscanner.medschl.cam.ac.uk/). We then matched the SNPs to the dataset of each IMID and filtered them under established conditions step-by-step before using them as IVs. For example, in SLE, we identified 12 SNPs significantly associated with circulating IL-18 levels were available in the SLE dataset. No proxy SNP was available in high LD (*r*^*2*^ > 0.8) with the specified genetic variant of interest. 2 SNPs were ambiguous and palindromic (with minor allele frequency threshold up to 0.3) and therefore excluded. Finally, 10 SNPs were used as IVs for the causality analysis between circulating IL-18 levels and SLE. We also calculated *R*^2^ using the “add_rsq” option of the TwoSampleMR package based on beta, standard error, and sample size to determine the variance explained for IL-18. The strategy outlined above for selecting genetic variants was repeated for analysing the associations with RA, IBD, AS, and PsO. Shown was a flow diagram reporting the procedure for IV selection in Supplementary Figure S1. The details of all eligible SNPs for final MR analyses were listed in Supplementary Tables S3 and S4.

### Mendelian randomization and statistical analysis

In the current study, we performed two-sample MR that reused data from relatively independent GWASs for exposure and outcomes to infer the causalities between IL-18 levels and the risk of the IMIDs. We applied the inverse variance-weighted (IVW) method under a multiplicative random-effects model to conduct primary MR analyses and generate the estimates for each outcome [24]. This method combined the Wald ratio estimates of individual SNP to generate the causal estimate for the MR association, where we calculated the ratio of SNP–outcome over SNP–exposure associations to obtain each estimate. The results were expressed as ORs on disease risk per one standard deviation unit increase along with the elevation of IL-18 levels.

Since the IVW estimates can be biased if pleiotropic instrumental variables are used, we also applied Weighted median (requiring that at least 50% of IVs are valid) [25], Robust adjusted profile score (RAPS) (providing a robust inference in the presence of weak IVs) [26], and Maximum likelihood (assuming that heterogeneity and horizontal pleiotropy do not exist) [27] methods for sensitivity analyses based on different assumptions. These methods are relatively less statistically powerful than IVW but can adjust for pleiotropy and assess the validity of MR findings. Since MR Egger is statistically less efficient (providing wider confidence intervals), especially if the number of genetic instruments is low, we only used MR Egger intercepts to verify our results in the aspect of the horizontal pleiotropy (a non-zero intercept with *P* < 0.05 indicated the presence of horizontal pleiotropy) [28]. We also quantified the heterogeneities across the SNPs by using Cochran’s Q statistic (*P* < 0.05 was considered significant) [29]. MR-Pleiotropy RESidual Sum and Outlier (MR-PRESSO) analysis was used to detect and correct potential horizontal pleiotropic outliers [30]. Furthermore, we inspected visual asymmetry in funnel plots and conducted the leave-one-out analysis to assess the influence of a single SNP on the observed associations.

Associations were considered statistically significant with an adjusted *P*-value after Bonferroni correction (*P* < 0.05/5 = 0.01, correcting for one exposure and five outcomes). For IMIDs nominally associated with IL-18, we performed reverse MR analysis to examine whether genetic predisposition to IMIDs affects IL-18 levels.

All statistical analyses and data visualization were performed in R version 4.3.1 (R Foundation for Statistical Computing, Vienna, Austria) using the following packages: TwoSampleMR (version 0.5.7) [31], ggplot2 [32], and forestplot [33].

## Results

### Mendelian randomization results

**Fig. 1:**
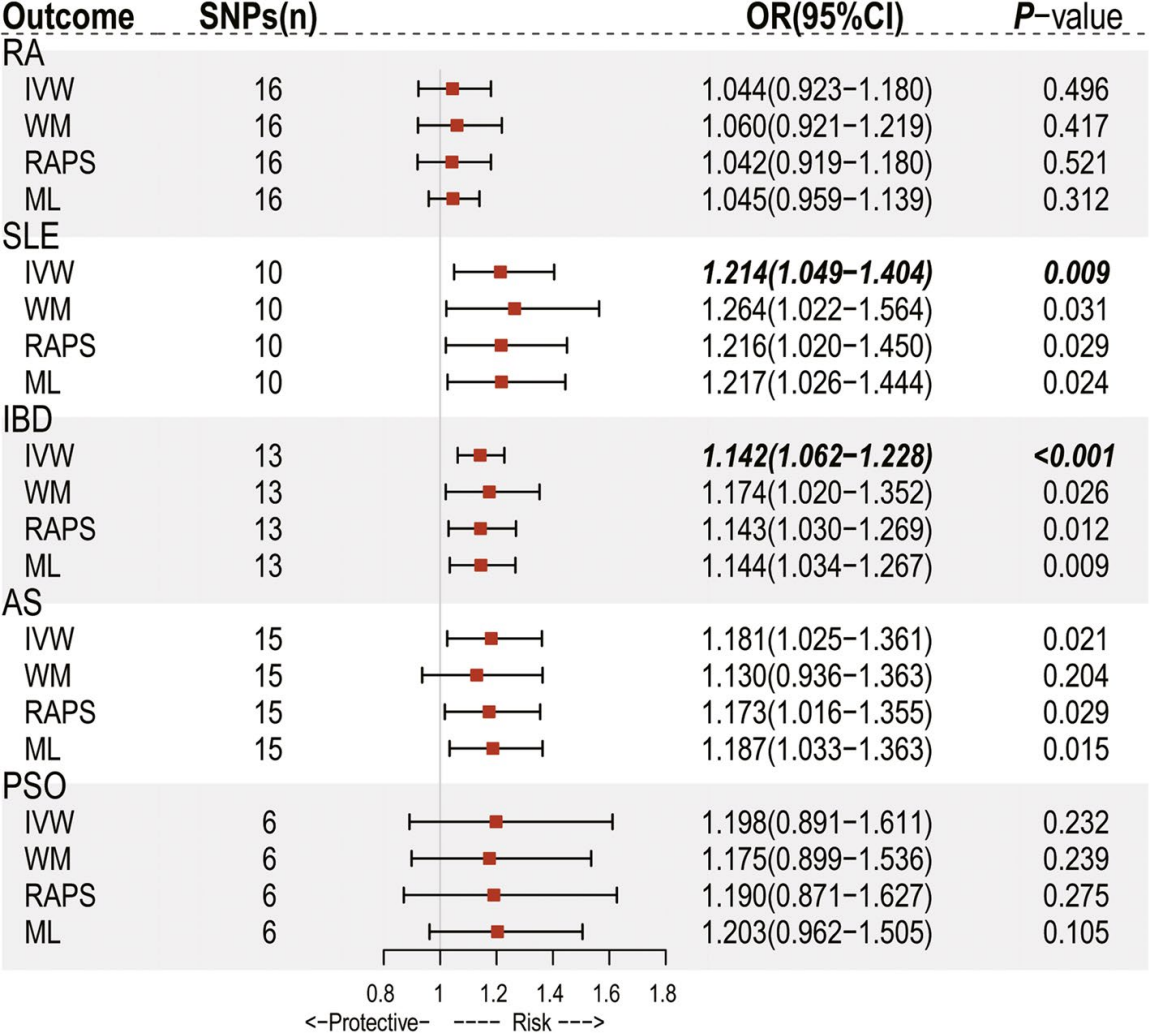
Forest plot of Mendelian randomization analyses for the associations of circulating levels of interleukin-18 with risk of five immune-mediated inflammatory diseases. CI, confidence interval; OR, odds ratio; SNP, single nucleotide polymorphism

After screening following the rules mentioned in Methods, 6 to 16 SNPs were selected as IVs and explained 2.78–7.55% of the variance (value of *R*^*2*^ statistic) for IL-18 levels in each IL18-IMID association (Supplementary Table S3 and S4). The *F* statistics for the selected instrument SNPs were no less than 30 (well above the threshold of *F* > 10 typically recommended for MR analyses), indicating that the IVs were strong enough to reduce the possibility of weak IV bias. Using the IVW method, we performed primary analyses and revealed a risk-causal effect for IL-18 levels on the risk of SLE and IBD, as depicted in Fig. 1. Specifically, the genetically elevated circulating IL-18 levels (1 SD increase) were causally associated with 21.4% higher odds for the risk of SLE (odds ratio [OR] = 1.214; 95% confidence interval [CI] = 1.049–1.404; *P* = 0.009) and with 14.2% higher odds for the risk of IBD (OR = 1.142; 95% CI = 1.062 − 1.228; *P* < 0.001). A suggestive significant association was found for AS (OR = 1.181; 95% CI = 1.025–1.361, *P* = 0.021). However, no significant risk or protective causal associations existed between genetically determined IL-18 levels and RA (OR = 1.044; 95% CI = 0.923–1.180, *P* = 0.496) or PsO (OR = 1.198; 95% CI = 0.891 − 1.611, *P* = 0.232).

### Sensitivity analyses

**Fig. 2:**
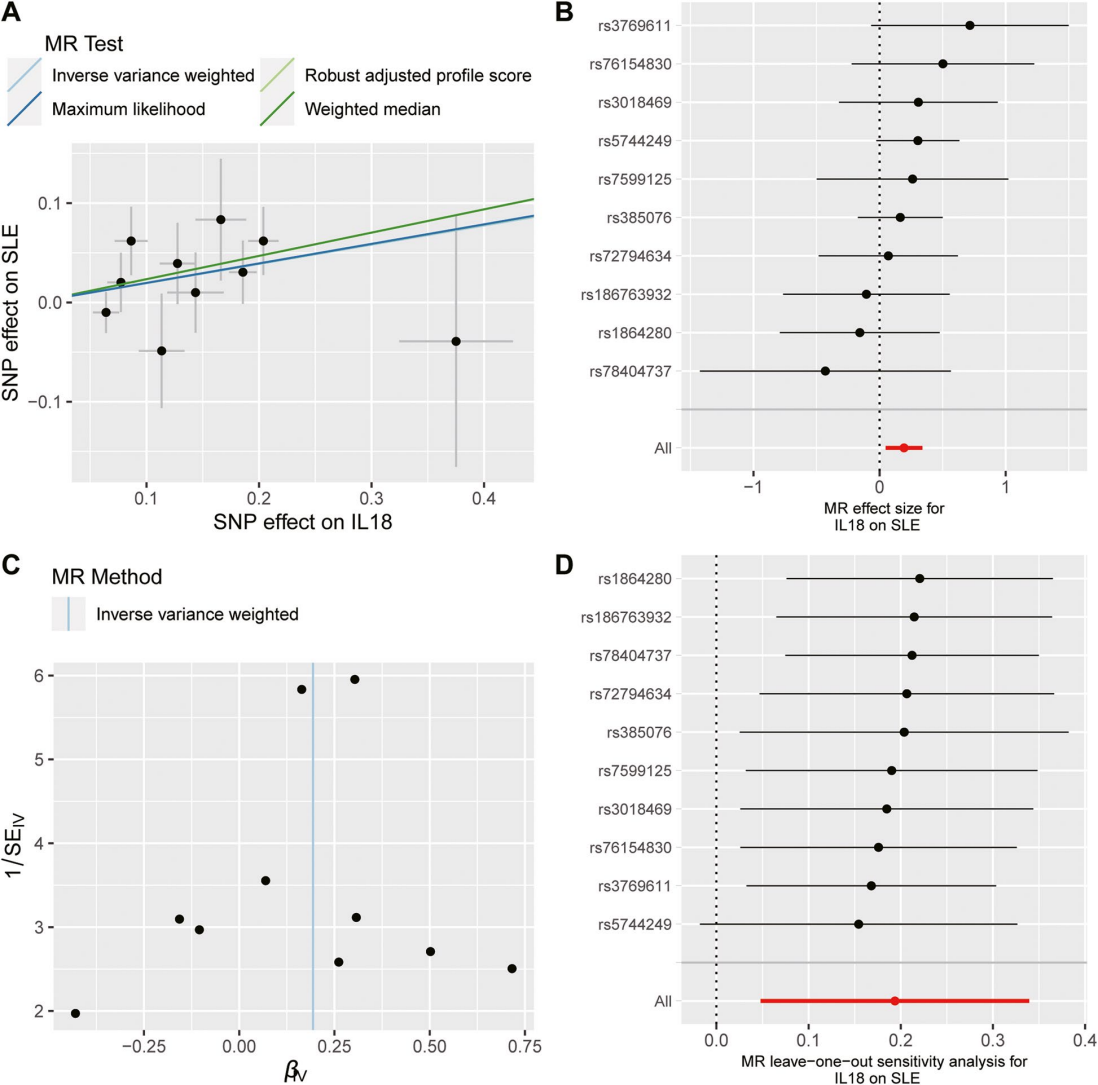
Analysis of the causal effect of increased circulating IL-18 levels on the risk of SLE. **A** Scatter plot of genetic association with circulating IL-18 levels (1 SD increase) against the genetic associations with SLE. The slope of the line represents the causal association, with each method having a different line. **B** Forest plot of the causal effects of circulating IL-18 levels (1 SD increase) on the risk of SLE. The causal effect of circulating IL-18 levels on SLE is estimated using each SNP singly (using the Wald ratio). The MR estimate using all SNPs derived from the IVW methods is shown for comparison. **C** Funnel plots of individual variant effects for the instrument variables plotted against the inverse of their standard error. **D** Leave-one-out analysis. Each dot in the forest plot represents the MR estimate (using IVW) excluding that particular SNP. The overall analysis (using IVW) including all SNPs is shown for comparison. IL-18, interleukin-18; SLE, systematic lupus erythematosus; SNP, single-nucleotide polymorphism; MR, Mendelian randomization; IVW, inverse variance weighted

**Fig. 3:**
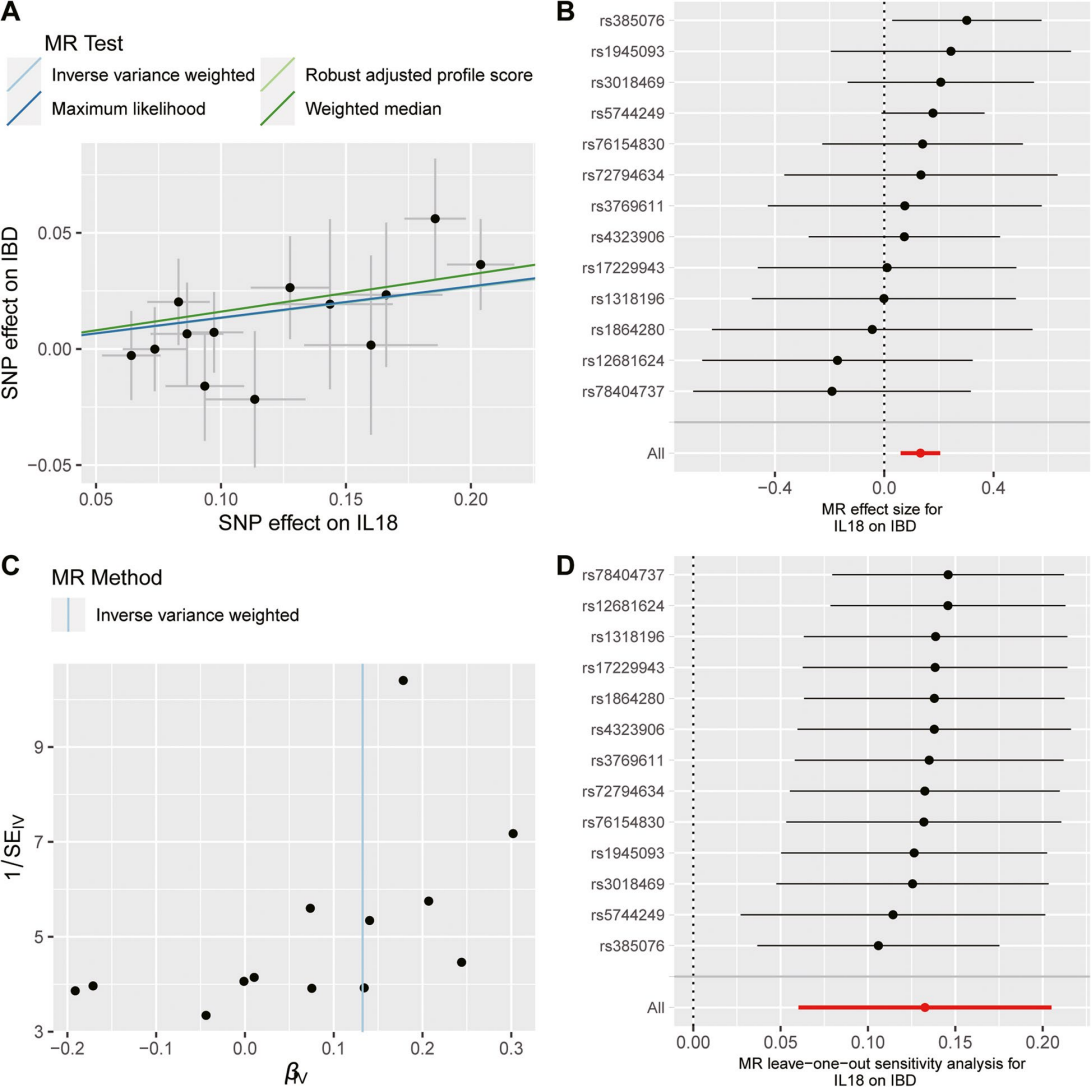
Analysis of the causal effect of increased circulating IL-18 levels on the risk of IBD. **A** Scatter plot of genetic association with circulating IL-18 levels (1 SD increase) against the genetic associations with IBD. The slope of the line represents the causal association, with each method having a different line. **B** Forest plot of the causal effects of circulating IL-18 levels (1 SD increase) on the risk of IBD. The causal effect of circulating IL-18 levels on SLE is estimated using each SNP singly (using the Wald ratio). The MR estimate using all SNPs derived from the IVW methods is shown for comparison. **C** Funnel plots of individual variant effects for the instrument variables plotted against the inverse of their standard error. **D** Leave-one-out analysis. Each dot in the forest plot represents the MR estimate (using IVW) excluding that particular SNP. The overall analysis (using IVW) including all SNPs is shown for comparison. IL-18, interleukin-18; IBD, inflammatory bowel disease; SNP, single-nucleotide polymorphism; MR, Mendelian randomization; IVW, inverse variance weighted

Sensitivity analysis using other MR methods yielded the results derived from the IVW method (Fig. 1). The Weighted median, RAPS, and Maximum likelihood demonstrated the same magnitude as the IVW meta-analysis, with OR ranging from 1.216 to 1.264 for the IL-18-SLE association and 1.143- 1.174 for the IL-18-IBD association. Cochran’s Q test showed no evidence for heterogeneity, and MR-Egger intercept analysis did not indicate any horizontal pleiotropy for the IVs (*P*_Cochran’s Q_ = 0.669 and *P*_intercept_ = 0.766 for the IL-18-SLE association; *P*_Cochran’s Q_ = 0.905 and *P*_intercept_ = 0.215 for the IL-18-IBD association). Analyses by the method of MR-PRESSO revealed no outliers (Table 1). We also plotted scatterplots and forest plots for the estimated associations, which visually ruled out the potential influence of outliers (Figs. 2 and 3). Notably, the leave-one-out analysis, by which we further systematically removed each SNP and repeated the MR analyses to obtain the influence of individual SNPs on the overall causal estimate, showed that the estimated effect for IL-18-SLE association was disproportionately influenced by rs5744249. The result for the IL-18-AS association derived from IVW was not robust when estimated using the Weighted median method. For IL-18-RA association, a potential heterogeneity could exist, but no significant horizontal pleiotropic outlier was identified in further MR-PRESSO outlier tests.

**Table 1:** Results of analyses for heterogeneity, horizontal pleiotropy and outliers

Exposure	Outcome	*P* for Cochran’s Q	*P* for MR-Egger intercept	*P* for MR-PRESSO global test
IL-18	RA	0.008	0.868	0.019*
	SLE	0.669	0.766	0.711
	IBD	0.905	0.215	0.906
	AS	0.382	0.495	0.417
	PSO	0.107	0.939	0.222
SLE	IL-18	0.267	0.255	0.814
IBD		0.770	0.608	0.786

*MR* Mendelian randomization, *MR-PRESSO* MR-Pleiotropy Residual Sum and Outlier, *IL-18* interleukin-18, *RA* rheumatoid arthritis, *SLE* systematic lupus erythematosus, *IBD* inflammatory bowel disease, *AS* ankylosing spondylitis, *PsO* psoriasis

^*^No significant outliers were identified

### Reverse MR analyses

**Fig. 4:**
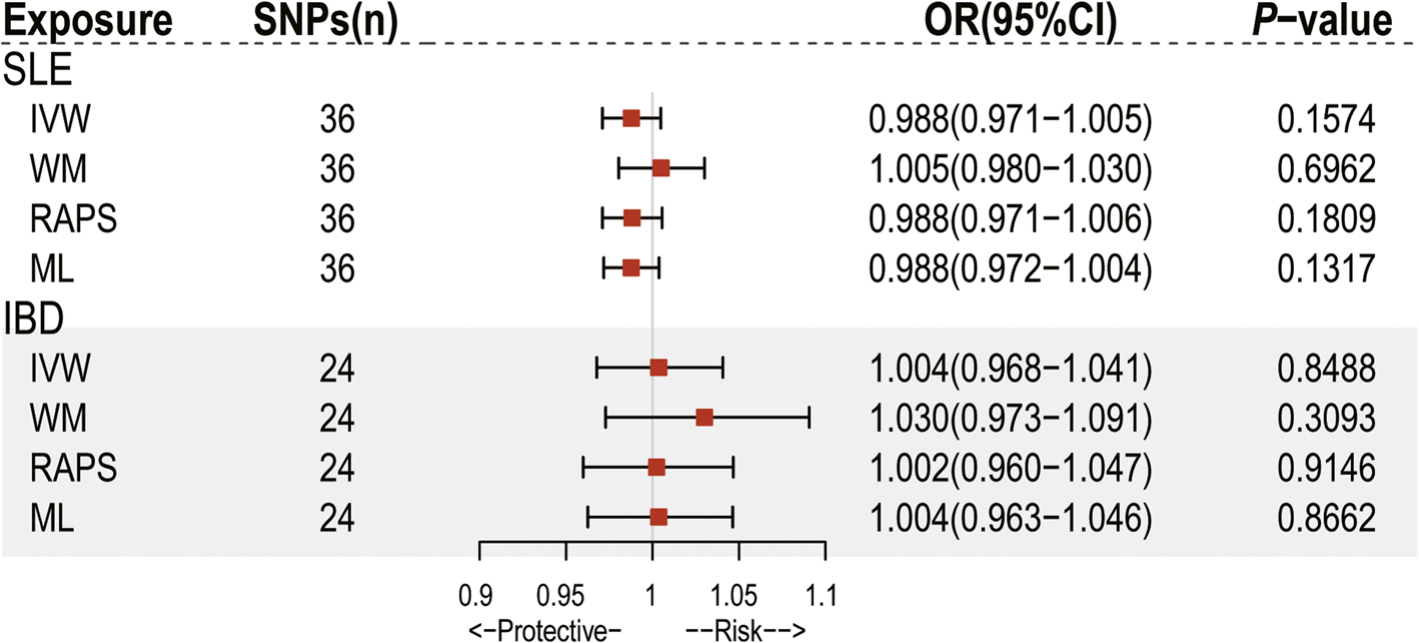
Forest plot of Mendelian randomization analyses for the associations of systematic lupus erythematosus and inflammatory bowel disease with circulating levels of interleukin-18. CI, confidence interval; OR, odds ratio; SNP, single nucleotide polymorphism

We further conducted MR analyses to explore the causal roles of SLE and IBD for circulating IL-18 levels by treating SLE and IBD as the exposures and IL-18 as the outcome. Detailed information for the instrument SNPs selected as IVs was presented in Supplementary Table S5. As was depicted in Fig. 4, neither SLE nor IBD had a causal effect on IL-18 levels (OR = 1.014, 95% CI = 0.981 − 1.048, *P* = 0. 406 for the SLE-IL18 association; OR = 0.977, 95% CI = 0.921 − 1.036, *P* = 0.440 for the IBD-IL18 association). The results were not influenced by bias since Cochran’s Q statistics *P* values, MR-Egger intercepts, and MR-PRESSO global results did not suggest any evidence of heterogeneity, horizontal pleiotropy, or outliers (Table 1, *P* > 0.05 for all). Scatterplots and forest plots showed no potential outlier that could affect the causal associations (Fig. S5 and S6). The results of the leave-one-out analysis (Fig. S5 and S6) demonstrated that no SNP potentially drove the causal relationships, implying our conclusion was stable.

## Discussion

IL-18 has been proven related to several IMIDs, but whether it exerts any causal effects remains unclear. In this study, by implementing two-sample MR analyses based on the GWAS data, we provide evidence supporting causal associations between genetically determined increased levels of circulating IL-18 and a higher risk of SLE and IBD. These findings were robust to pleiotropy and heterogeneity and stable in alternative MR methods.

Our results are consistent with previous human and animal studies demonstrating that IL-18 expression is higher in SLE and positively correlates with disease activity. A large amount of evidence from animal models and human diseases supports the role of IL-18 in SLE [34–36]. Serum IL-18 levels in MRL/lpr mice are higher compared with control animals. Administration of exogenous IL-18 to these mice aggravated disease activity and nephritis, and IL-18-deficient mice or mice treated with anti-IL-18 in the MRL/lpr model showed improved survival rates and reduction of proteinuria [13, 37–39]. In human studies, serum IL-18 levels in SLE patients are higher than in healthy controls. Some reports say that serum IL-18 levels are positively correlated with SLE disease activity, with others reporting IL-18 being associated with active renal disease [40–44]. Although the exact biological role of IL-18 in the pathogenesis of SLE remains unclear, there are several plausible explanations. First, IL-18, previously known as IFN-γ-inducing factor (IGIF), is an effective activator of polarized Th1 cells. A central pathological effect has been described for the inflammatory Th1-dependent cytokine IFN-γ. In synergy with other Th1-related cytokines, IL-18 will amplify the production of IFN-γ [45]. Second, IL-18 can stimulate the production of inflammatory cytokines, such as TNF-α and IL-1β, in mature Th1 cells, monocytes/macrophages, and NK cells. It can further up-regulate the expression of chemokines and adhesion molecules, enhance cytotoxicity, and induce the release of matrix metalloproteinases. All the activities are central to the inflammatory response and subsequent tissue damage [46]. Third, both Th1 and Th2 cells are involved in the pathogenesis of SLE. Th1 cells are pivotal for systemic nephritis, while Th2 cells are related to facial rash [47]. IL-18 is a unique cytokine that can induce Th1 or Th2 polarization depending on the immunologic context, thus being essential for the adequate Th1-dependent response and the balance with Th2 response [48]. The current study showed that rs5744249, which is located in the intron of the IL18 gene, could potentially drive the causal relationship between IL-18 and SLE. Since we had excluded the horizontal pleiotropy and weak instrument to a great extent, this result of leave-one-out analysis implied a vital biological impact of this SNP on SLE. As the abnormal production of cytokines plays a critical role in SLE by orchestrating the immune activation, targeting specific inflammatory cytokines could reduce SLE risk. However, results from clinical studies have supported the successful clinical translation of treatments targeting only a few cytokines, like BAFF and IFN [49]. To date, no clinical trial targeting IL-18 on SLE has been conducted. Therapeutic strategies aimed at blocking overexpressed IL-18 may be promising for treating SLE and are worthy of further determination in more studies.

Our results also supported that elevated IL-18 levels are causally associated with an increased risk of IBD, consistent with the previously published MR study [50]. Even though Mokry et al. had already conducted a comprehensive study, our study can strengthen the previous results and use IBD as a positive control in the current analyses, with a larger dataset for IBD, more feasible terms for screening IVs, and different MR methods. Multiple studies have reported elevated levels of IL-18 in the serum and intestinal tissues of individuals with IBD [8, 51–53], and genetic variations in the IL-18 gene were associated with an increased risk of IBD [54]. In animal studies, increased expression of IL-18 was linked to exacerbation of colitis, and blocking IL-18 signalling can reduce inflammation and alleviate symptoms in models of IBD [55], highlighting the importance of IL-18 as a target for new drugs for IBD treatment. A phase II trial of a recombinant human IL-18-neutralizing antibody for treating moderate to severe Crohn's disease (CD) is currently under testing (ClinicalTrials.gov Identifier: NCT03681067).

In contrast, our results provided no robust evidence for a causal association between IL-18 and RA, AS, or PsO. In coordination with this, a first clinical trial using tadekinig alfa, a human recombinant IL-18 binding protein (BP), in patients with RA and PsO failed to demonstrate therapeutic efficacy [56] despite the elevated circulating levels and tissue expression of IL-18 has been described in these diseases [12, 57–64]. A possible explanation for this is that previous studies used immunoassays that could not differentiate between IL-18 bound to IL-18BP and free bioactive IL-18, which limits interpretation regarding the role of IL-18 in these diseases. As IL-18BP has a high IL-18 sequestration capacity [47], the balance between IL-18/IL-18BP and the concentrations of free IL-18, instead of total IL-18, is more relevant to measure to evaluate inflammatory responses [65]. Remarkably, free IL-18 levels were not higher in RA or AS compared to healthy individuals [66], whereas free IL-18 levels remain significantly higher in SLE and CD patients than in controls despite the overproduction of IL-18BP [47, 67–69].

The predominant advantage of this study is that the MR design allows a comprehensive assessment of the relations between genetically predicted circulating IL-18 levels and five common IMIDs in independent European populations simultaneously. Genetic correlation helps us to explain the diverse relationships between IL-18 and IMIDs from the view of shared genetic risk. The results were confirmed through sensitivity analyses for pleiotropy, including alternative MR methods. This study restricted to European populations could minimize bias by population stratification; however, it might limit the generalizability of our findings to other races or ethnicities.

Some limitations need to be considered when interpreting the results of this study. There may be sample overlap between exposure data and outcome data, which can lead to model overfitting and causal estimates biased towards observed estimates (type 1 error) [70]. However, the sample overlap is less likely to mislead our results, as our SNPs were selected from large-scale GWASs at a genome-wide threshold (strongly associated with exposure), and all estimated *F* statistics exceeded 10. Moreover, there might be interaction effects among the IMIDs, for example, IBD and AS, which could not be assessed in this MR analysis based on summary-level data. The results in the current study should be addressed further in future studies with refined design. Also, genetically determined IL-18 measures lifelong IL-18 levels and only acquires population-averaged effects rather than short-term responses to inflammation. Future MR studies are warranted to assess the non-linear association of IL-18 levels with the disease risk of IMIDs. Last but not least, the differences regarding the subgroups of IBD and diverse clinical manifestations of SLE are essential since IL-18 is likely to play distinct roles. Although IL-18 was not associated with PsO, there may remain a causal association of IL-18 for specific subtypes of PsO to be found.

## Conclusions

This MR study provided evidence that genetically determined elevated IL-18 levels were causally associated with risks of SLE and IBD but not RA, AS, or PsO. Our results supported the blocking IL-18 treatment for IBD and suggested that targeting IL-18 could be a promising therapeutic strategy for treating SLE. Future studies should focus on elucidating the precise role of IL-18 in these diseases.

### Supplementary Information

**Additional file 1: Supplementary Table S1.** Strengthening the Reporting of Observational Studies in Epidemiology using Mendelian Randomization (STROBE-MR) checklist. **Supplementary Table S2.** Baseline characteristics of the study population. **Supplementary Table S3.** Information of genetic variants used as instrument variables for interleukin-18 levels. **Supplementary Table S4.** Information for instrument variables of IL-18 levels in each immune-mediated inflammatory disease. **Supplementary Table S5.** Genetic variants as instrument variables for systemic lupus erythematosus and inflammatory bowel disease with interleukin-18 levels.

**Additional file 2:** **Fig. S1. **Flow chart for instrumental variable (IV) selection process. **Fig. S2. **Analysis of the causal effect of increased circulating IL-18 levels on the risk of RA. **Fig. S3. **Analysis of the causal effect of increased circulating IL-18 levels on the risk of AS. **Fig. S4. **Analysis of the causal effect of increased circulating IL-18 levels on the risk of PsO. **Fig. S5. **Analysis of the causal effect of risk of SLE on circulating IL-18 levels. **Fig. S6. **Analysis of the causal effect of risk of IBD on circulating IL-18 levels.  

## Data Availability

The original summary statistics can be obtained from the published GWASs or Risteys FinnGen R9 (https://r9.finngen.fi/).

## Abbreviations

AS: Ankylosing spondylitis
SLE: Systemic lupus erythematosus
RA: Rheumatoid arthritis
IBD: Inflammatory bowel disease
PsO: Psoriasis
IL: Interleukin
MR: Mendelian randomization
GWAS: Genome-wide association study
SNPs: Single-nucleotide polymorphisms
LD: Linkage disequilibrium
IVs: Instrumental variables
IVW: Inverse variance weighted
MR-RAPS: MR-robust adjusted profile score
MR-PRESSO: MR- Pleiotropy Residual Sum and Outlier
OR: Odds ratio
CI: Confidence interval
IFN: Interferon
BAFF: B cell-activating factor
