# Supplementary material for: Evaluation of causal associations between interleukin-18 levels and immune-mediated inflammatory diseases: a Mendelian randomization study
Source: BMC Med Genomics. 2023 Nov 29;16:306. doi: 10.1186/s12920-023-01744-z (PMC10685486; doi:10.1186/s12920-023-01744-z)
Supplement: Supplementary file 2 — Additional file 2: Fig. S1. Flow chart for instrumental variable (IV) selection process. Fig. S2. Analysis of the causal effect of increased circulating IL-18 levels on the risk of RA. Fig. S3. Analysis of the causal effect of increased circulating IL-18 levels on the risk of AS. Fig. S4. Analysis of the causal effect of increased circulating IL-18 levels on the risk of PsO. Fig. S5. Analysis of the causal effect of risk of SLE on circulating IL-18 levels. Fig. S6. Analysis of the causal effect of risk of IBD on circulating IL-18 levels. [file 12920_2023_1744_MOESM2_ESM.pdf]

**Fig. S1**

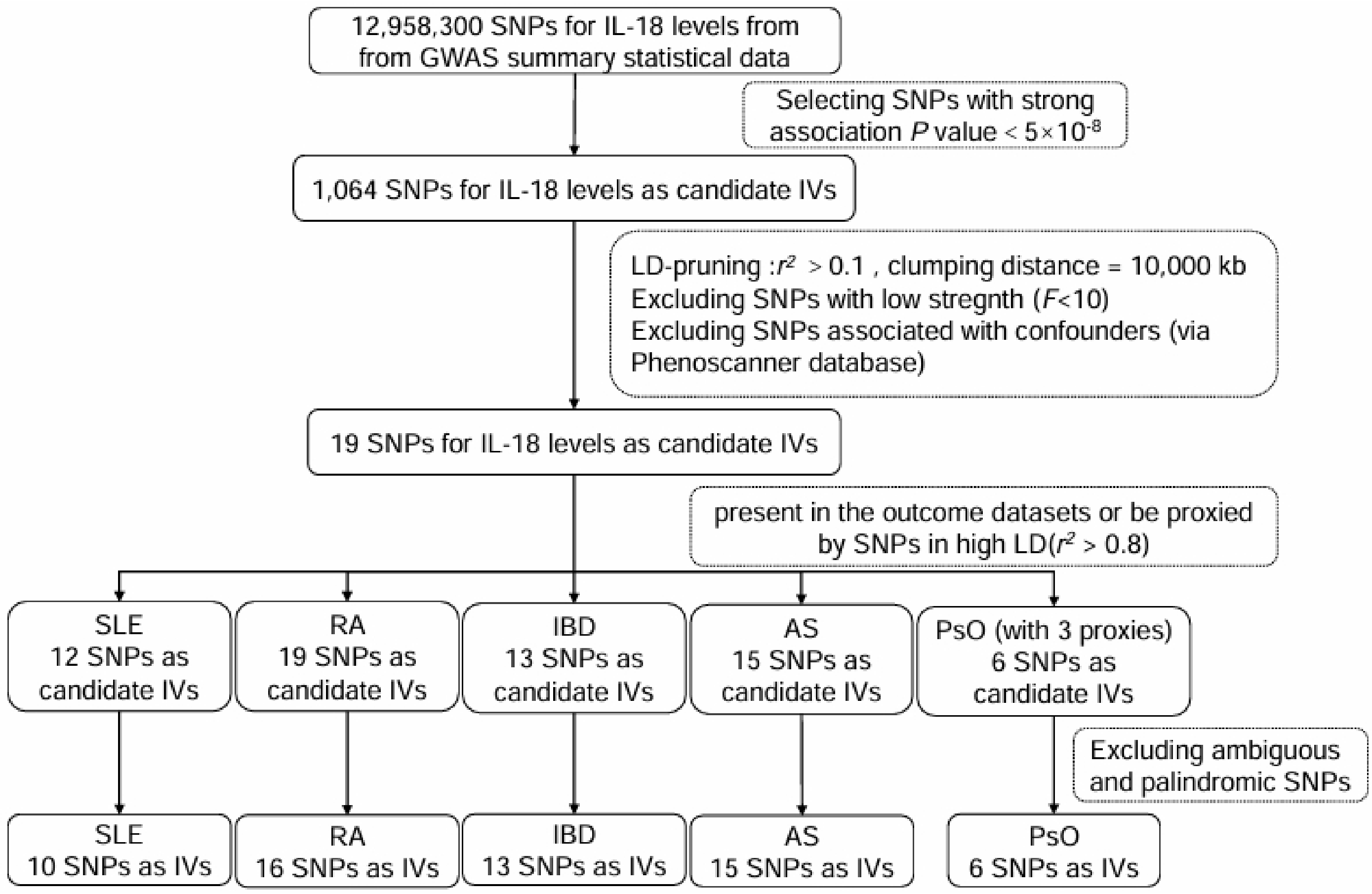
12,958,300 SNPs for IL-18 levels from
from GWAS summary statistical data
Selecting SNPs with strong association P value < 5×10-8
1,064 SNPs for IL-18 levels as candidate IVs
LD-pruning :r2 > 0.1 , clumping distance = 10,000 kb
Excluding SNPs with low stregnth (F<10)
Excluding SNPs associated with confounders (via Phenoscanner database)
19 SNPs for IL-18 levels as candidate IVs
present in the outcome datasets or be proxied by SNPs in high LD(r2 > 0.8)
SLE 12 SNPs as candidate IVs
RA 19 SNPs as candidate IVs
IBD 13 SNPs as candidate IVs
AS 15 SNPs as candidate IVs
PsO (with 3 proxies) 6 SNPs as candidate IVs
Excluding ambiguous and palindromic SNPs
SLE 10 SNPs as IVs
RA 16 SNPs as IVs
IBD 13 SNPs as IVs
AS 15 SNPs as IVs
PsO 6 SNPs as IVs

**Fig. S2**

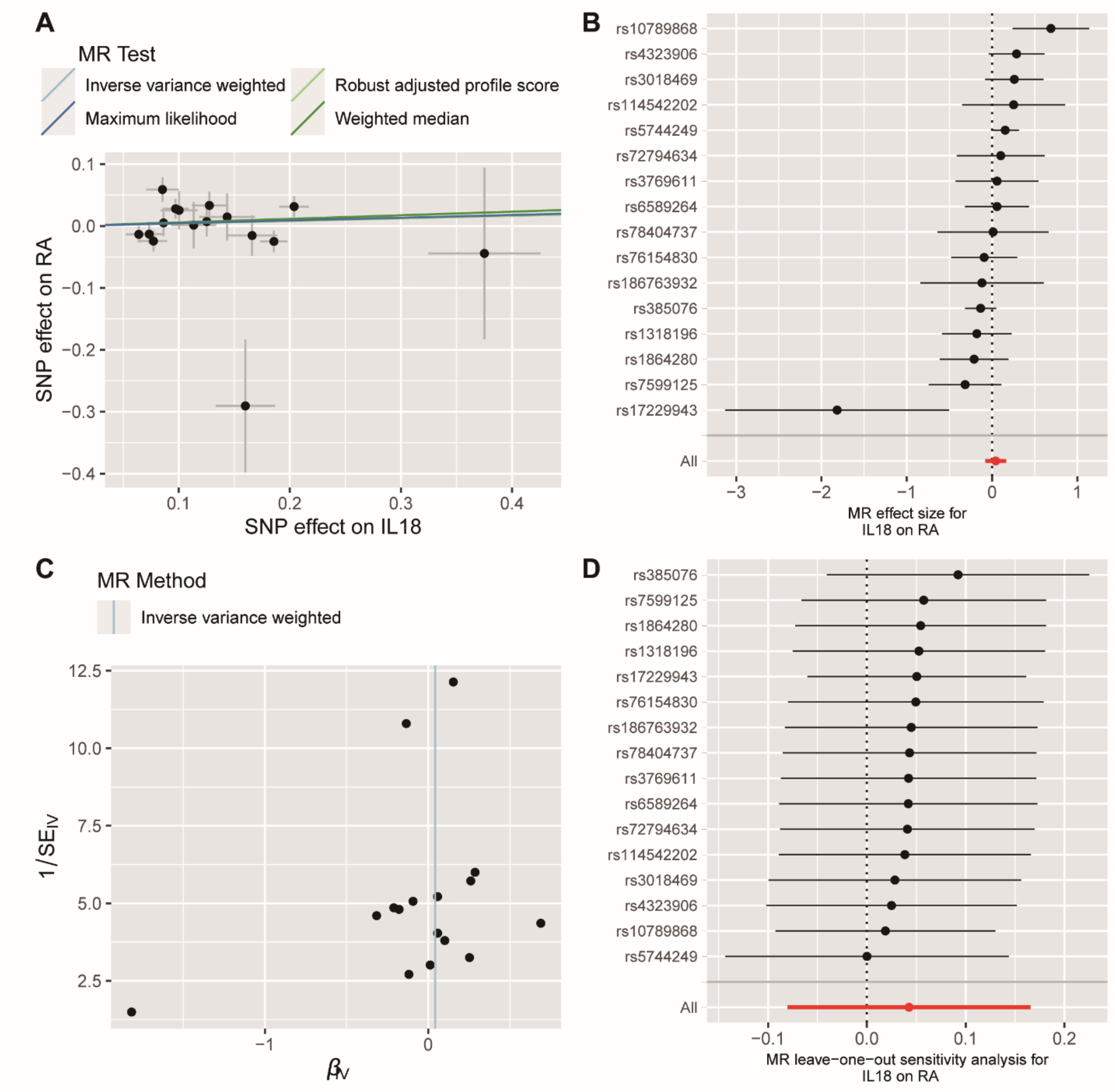
A
MR Test
Inverse variance weighted
Maximum likelihood
Robust adjusted profile score
Weighted median
SNP effect on RA
SNP effect on IL18
0.1
0.0
−0.1
−0.2
−0.3
−0.4
0.1
0.2
0.3
0.4
B
rs10789868
rs4323906
rs3018469
rs114542202
rs5744249
rs72794634
rs3769611
rs6589264
rs78404737
rs76154830
rs186763932
rs385076
rs1318196
rs1864280
rs7599125
rs17229943
All
−3
−2
−1
0
1
MR effect size for
IL18 on RA
C
MR Method
Inverse variance weighted
12.5
10.0
7.5
5.0
2.5
1/SE$_{IV}$
−1
0
$\beta_{IV}$
D
rs385076
rs7599125
rs1864280
rs1318196
rs17229943
rs76154830
rs186763932
rs78404737
rs3769611
rs6589264
rs72794634
rs114542202
rs3018469
rs4323906
rs10789868
rs5744249
All
−0.1
0.0
0.1
0.2
MR leave−one−out sensitivity analysis for
IL18 on RA

Fig. S3

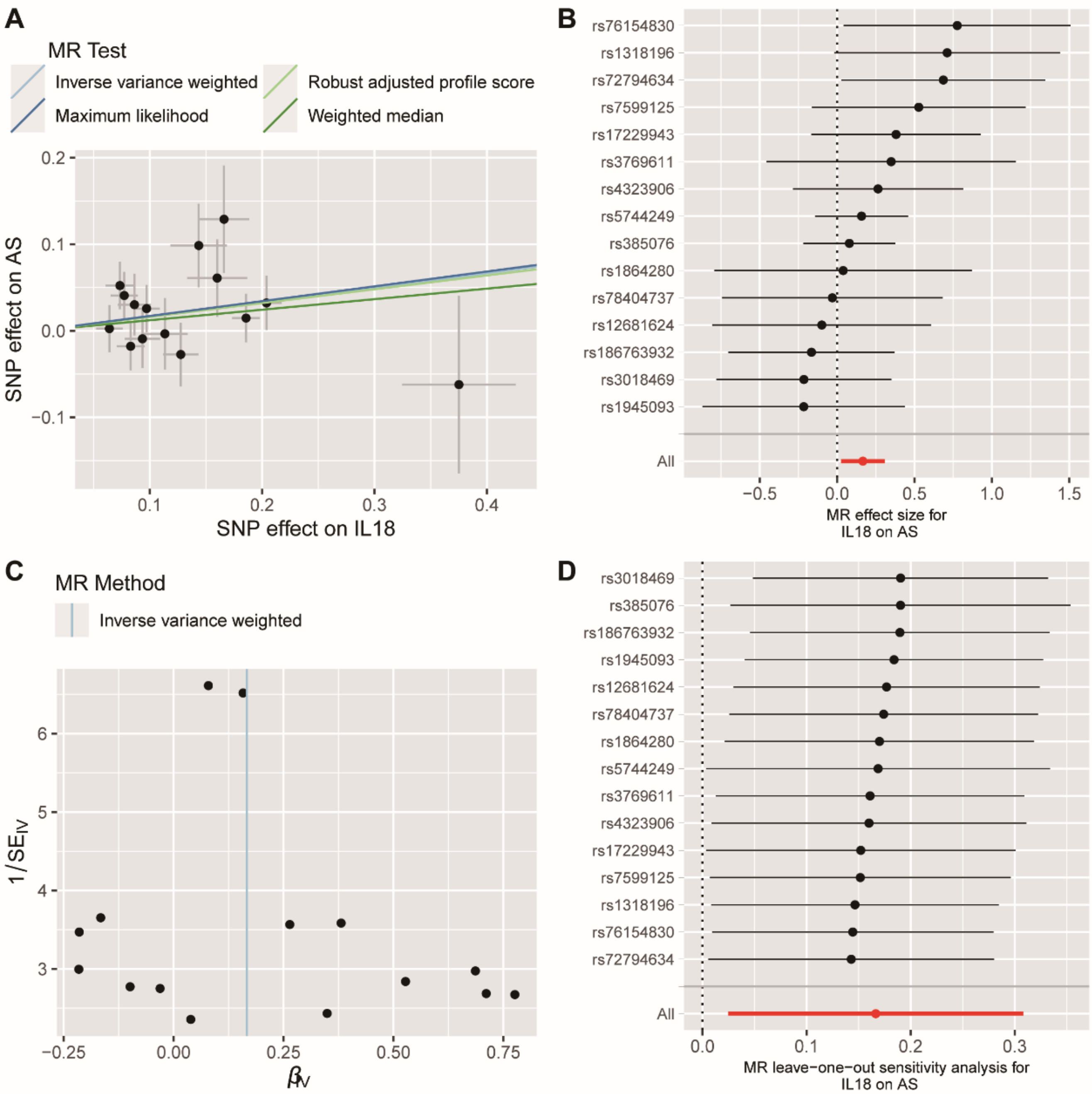

A
MR Test
Inverse variance weighted
Maximum likelihood
Robust adjusted profile score
Weighted median
SNP effect on AS
SNP effect on IL18
B
rs76154830
rs1318196
rs72794634
rs7599125
rs17229943
rs3769611
rs4323906
rs5744249
rs385076
rs1864280
rs78404737
rs12681624
rs186763932
rs3018469
rs1945093
All
MR effect size for IL18 on AS
C
MR Method
Inverse variance weighted
1/SE_IV
β_IV
D
rs3018469
rs385076
rs186763932
rs1945093
rs12681624
rs78404737
rs1864280
rs5744249
rs3769611
rs4323906
rs17229943
rs7599125
rs1318196
rs76154830
rs72794634
All
MR leave-one-out sensitivity analysis for IL18 on AS

**Fig. S4**

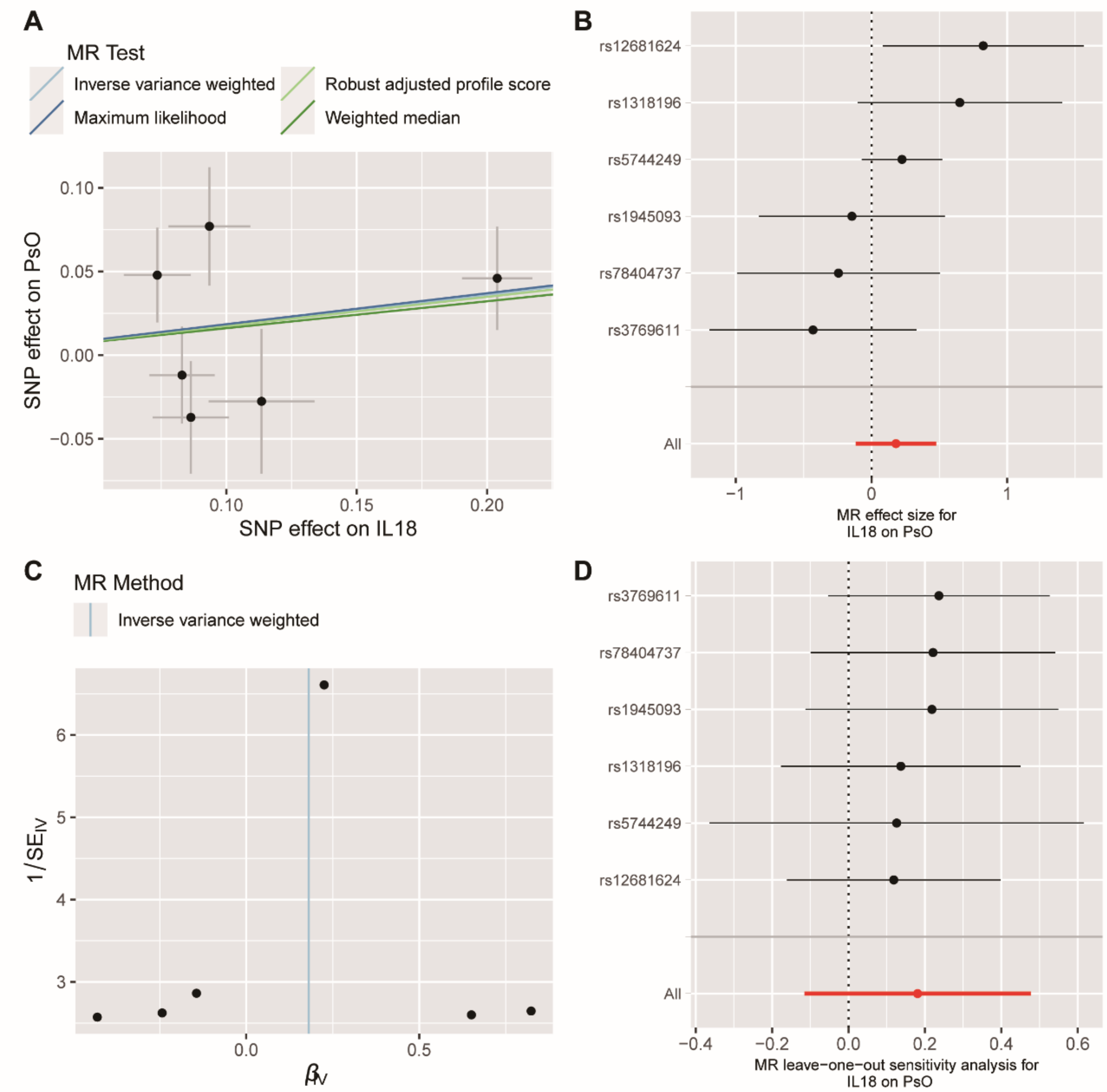
A
MR Test
Inverse variance weighted
Maximum likelihood
Robust adjusted profile score
Weighted median
SNP effect on PsO
SNP effect on IL18
B
rs12681624
rs1318196
rs5744249
rs1945093
rs78404737
rs3769611
All
MR effect size for
IL18 on PsO
C
MR Method
Inverse variance weighted
1/SE_IV
β_IV
D
rs3769611
rs78404737
rs1945093
rs1318196
rs5744249
rs12681624
All
MR leave−one−out sensitivity analysis for
IL18 on PsO

**Fig. S5**

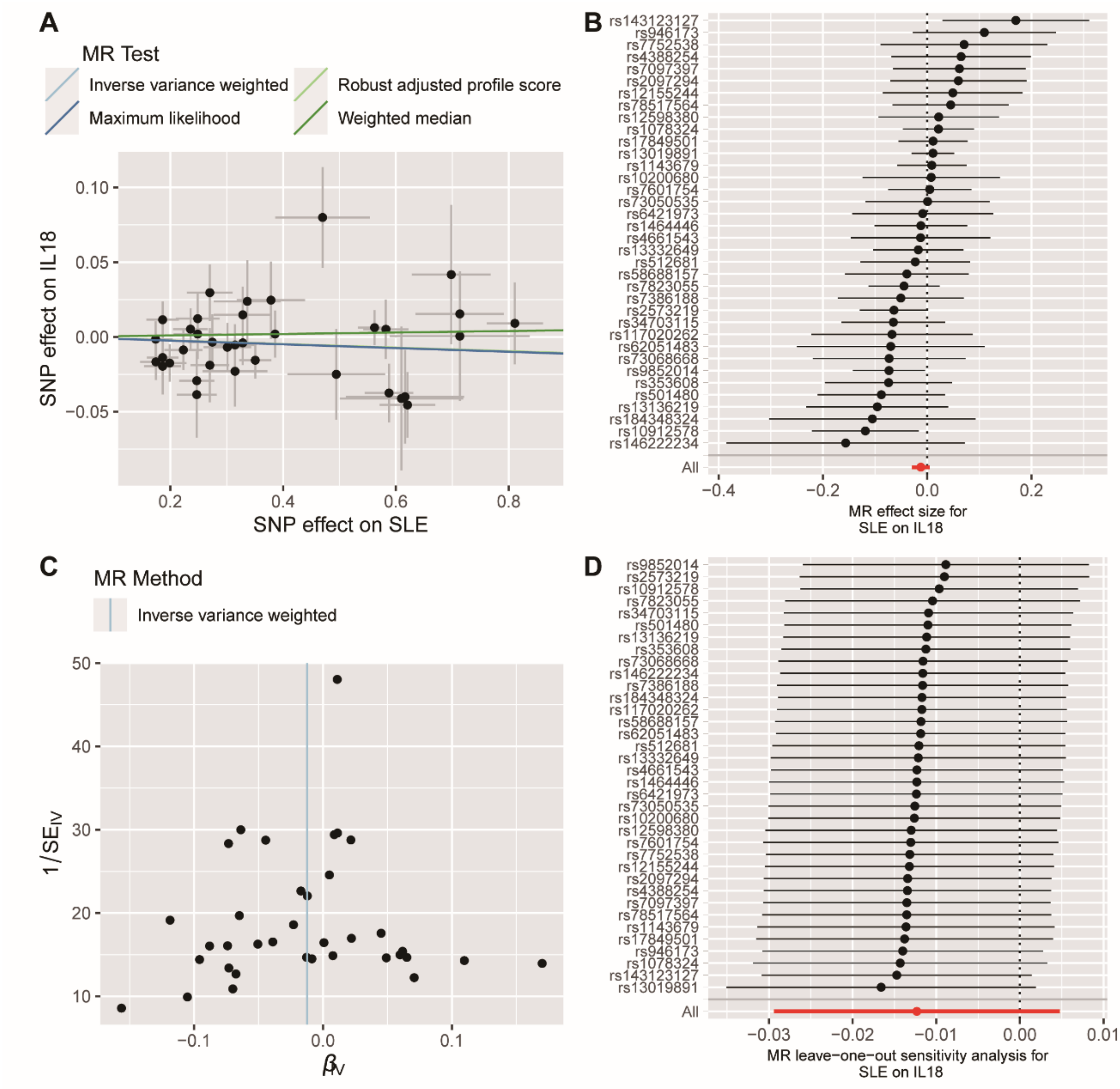

Fig. S6

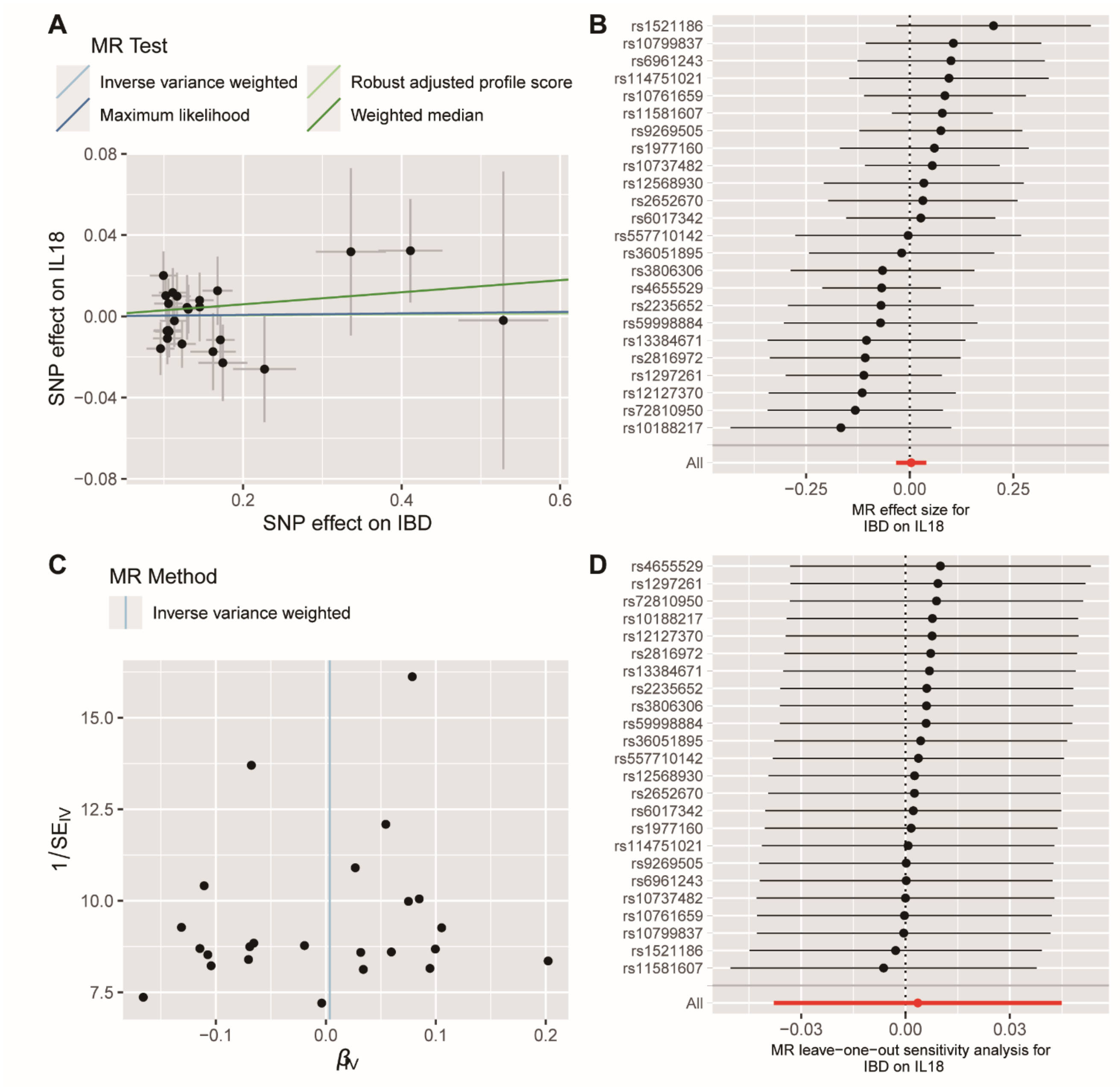


**Fig. S1 Flow chart for instrumental variable (IV) selection process.**

**Fig. S2 Analysis of the causal effect of increased circulating IL-18 levels on the risk of RA.** (A) Scatter plots of genetic associations with circulating IL-18 levels (1 SD increase) against the genetic associations with RA. The slope of the line represents the causal association, with each method having a different line. (B) Forest plot of the causal effects of circulating IL-18 levels (1 SD increase) on the risk of RA. The causal effect of circulating IL-18 levels on RA is estimated using each SNP singly (using the Wald ratio). The MR estimate using all SNPs derived from the IVW methods is shown for comparison. (C) Funnel plots of

individual variant effects for the instrument variables plotted against the inverse of their standard error. (D) Leave-one-out analysis. Each dot in the forest plot represents the MR estimate (using IVW) excluding that particular SNP. The overall analysis including all SNPs is shown for comparison. IL-18, interleukin-18; RA, rheumatoid arthritis; SNP, single-nucleotide polymorphism; MR, Mendelian randomization; IVW, inverse variance weighted.

**Fig. S3 Analysis of the causal effect of increased circulating IL-18 levels on the risk of AS.** (A) Scatter plots of genetic associations with circulating IL-18 levels (1 SD increase) against the genetic associations with AS. The slope of the line represents the causal association, with each method having a different line. (B) Forest plot of the causal effects of circulating IL-18 levels (1 SD increase) on the risk of AS. The causal effect of circulating IL-18 levels on AS is estimated using each SNP singly (using the Wald ratio). The MR estimate using all SNPs derived from the IVW methods is shown for comparison. (C) Funnel plots of individual variant effects for the instrument variables plotted against the inverse of their standard error. (D) Leave-one-out analysis. Each dot in the forest plot represents the MR estimate (using IVW) excluding that particular SNP. The overall analysis including all SNPs is shown for comparison. IL-18, interleukin-18; AS, ankylosing spondylitis; SNP, single-nucleotide polymorphism; MR, Mendelian randomization; IVW, inverse variance weighted.

**Fig. S4 Analysis of the causal effect of increased circulating IL-18 levels on the risk of PsO.** (A) Scatter plots of genetic associations with circulating IL-18 levels (1 SD increase) against the genetic associations with PsO. The slope of the line represents the causal association, with each method having a different line. (B) Forest plot of the causal effects of circulating IL-18 levels (1 SD increase) on the risk of PsO. The causal effect of circulating IL-18 levels on SLE is estimated using each SNP singly (using the Wald ratio). The MR estimate using all SNPs derived from the IVW methods is shown for comparison. (C) Funnel plots of individual variant effects for the instrument variables plotted against the inverse of their standard error. (D) Leave-one-out analysis. Each dot in the forest plot represents the MR estimate (using IVW) excluding that

particular SNP. The overall analysis including all SNPs is shown for comparison. IL-18, interleukin-18; PsO, psoriasis; SNP, single-nucleotide polymorphism; MR, Mendelian randomization; IVW, inverse variance weighted.

**Fig. S5 Analysis of the causal effect of risk of SLE on circulating IL-18 levels.** (A) Scatter plots of genetic associations with SLE against the genetic associations with circulating IL-18 levels (1 SD increase). The slope of the line represents the causal association, with each method having a different line. (B) Forest plot of the causal effects of risk of SLE on circulating IL-18 levels (1 SD increase). The causal effect of SLE on circulating IL-18 levels is estimated using each SNP singly (using the Wald ratio). The MR estimate using all SNPs derived from the IVW methods is shown for comparison. (C) Funnel plots of individual variant effects for the instrument variables plotted against the inverse of their standard error. (D) Leave-one-out analysis. Each dot in the forest plot represents the MR estimate (using IVW) excluding that particular SNP. The overall analysis including all SNPs is shown for comparison. SLE, systematic lupus erythematosus; IL-18, interleukin-18; SNP, single-nucleotide polymorphism; MR, Mendelian randomization; IVW, inverse variance weighted.

**Fig. S6 Analysis of the causal effect of risk of IBD on circulating IL-18 levels.** (A) Scatter plots of genetic associations with IBD against the genetic associations with circulating IL-18 levels (1 SD increase). The slope of the line represents the causal association, with each method having a different line. (B) Forest plot of the causal effects of risk of IBD on circulating IL-18 levels (1 SD increase). The causal effect of IBD on circulating IL-18 levels is estimated using each SNP singly (using the Wald ratio). The MR estimate using all SNPs derived from the IVW methods is shown for comparison. (C) Funnel plots of individual variant effects for the instrument variables plotted against the inverse of their standard error. (D) Leave-one-out analysis. Each dot in the forest plot represents the MR estimate (using IVW) excluding that particular SNP. The overall analysis including all SNPs is shown for comparison. IBD, inflammatory bowel disease; IL-18, interleukin-18; SNP, single-nucleotide polymorphism; MR, Mendelian randomization; IVW, inverse

variance weighted.
